# Modulation of LPS-Induced CD4+ T-Cell Activation and Apoptosis by Antioxidants in Untreated Asymptomatic HIV Infected Participants: An *In Vitro* Study

**DOI:** 10.1155/2013/631063

**Published:** 2013-11-21

**Authors:** S. Mburu, J. L. Marnewick, A. Abayomi, H. Ipp

**Affiliations:** ^1^Haematology Division, Department of Pathology, Faculty of Medicine and Health Science, Stellenbosch University, P.O. Box 19063, Tygerberg, Cape Town 7505, South Africa; ^2^Oxidative Stress Research Centre, Department of Biomedical Sciences, Faculty of Health and Wellness Sciences, Cape Peninsula University of Technology, P.O. Box 1906, Bellville 7535, South Africa

## Abstract

Persistent immune activation characterises HIV infection and is associated with depletion of CD4+ T-cells and increased risk of disease progression. Early loss of gut mucosal integrity results in the translocation of microbial products such as lipopolysaccharide (LPS) into the systemic circulation. This is an important source of on-going immune stimulation. The purpose of this study was to determine levels of CD4+ T-cell activation (%CD25 expression) and apoptosis (% annexin V/7-AAD) in asymptomatic, untreated HIV infection at baseline and after stimulation with LPS and incubation with or without vitamin C and N-acetylcysteine. LPS induced a significant (*P* < 0.03) increase in %CD25 expression, annexin V, and 7-AAD in HIV positive individuals. NAC in combination with vitamin C, significantly (*P* = 0.0018) reduced activation and early apoptosis of CD4+ T-cells to a greater degree than with either antioxidant alone. Certain combinations of antioxidants could be important in reducing the harmful effects of chronic immune activation and thereby limit CD4+ T-cell depletion. Importantly, we showed that CD4+ T-cells of the HIV positive group responded better to a combination of the antioxidants at this stage than those of the controls. Therefore, appropriate intervention at this asymptomatic stage could rescue the cells before repetitive activation results in the death of CD4+ T-cells.

## 1. Introduction

HIV infection is characterized by chronic immune activation and inflammatory cytokine production [1, 2]. The consistent activation of CD4 and CD8 T-cells is associated with depletion of CD4 T-cells and increased risk of disease progression to AIDS [3]. Furthermore, markers of immune activation have been shown to be stronger predictors of progression to AIDS than either the CD4 counts or viral loads [4–6]. In particular, increased T-cell activation has been associated with AIDS suggesting that activated T-cells are susceptible to apoptosis [7]. In addition, elevated levels of CD38, a marker of immune activation on CD4 and CD8 T-cell, predict a rapid decrease of CD4 T-cells and a shorter survival rate, independent of HIV viral loads [8, 9].

The significant depletion of memory-type CD4 T-cells lining the gastrointestinal tract (GIT) mucosa in early HIV infection results in the breakdown of the mucosa and on-going translocation of microbial products such as lipopolysaccharide (LPS) across the epithelial surface [1, 10]. LPS induces activation of innate immune cells such as monocytes and dendritic cells resulting in increased oxidative stress; depletion of antioxidant defence mechanisms and an increased susceptibility to apoptosis [1, 11]. In addition, CD4 T-lymphocytes have been shown to express-toll-like receptor-4 (TLR4), which is a receptor for LPS [12, 13]. Triggering of TLR4 activates various signalling pathways such as mitogen-activated protein kinases (MAPKs), p38, and JNK, which induce activation of transcription factor nuclear factor of kappa B (NF-*κ*B) and subsequent production of proinflammatory cytokines, chemokines, antimicrobial peptides, and other defence molecules such as ROS [13]. The proinflammatory cytokines such as TNF-*α*, are capable of activating innate immune cells to produce more ROS. ROS and proinflammatory cytokines in turn induce activation of both the extrinsic and intrinsic pathways of apoptosis. The role of *in vitro* stimulation with lipopolysaccharide (LPS) on T-cell activation in HIV has been explored only minimally [14, 15]. The first study to date in HIV used CD38 and HLA-DR as the activation markers which were conducted on HIV positive patients who were on antiretroviral treatment [15]. Few studies have investigated the inhibitory effects by antioxidants on immune activation and apoptosis in asymptomatic, untreated HIV infection. In the current study, we investigated the effects of vitamin C and NAC on LPS-induced upregulation of interleukin receptor-2 receptor alpha chain, (CD25), as a marker of LPS-induced activation of CD4+T-cells after overnight incubation in untreated HIV infection.

In this study, therefore, levels of immune activation and apoptosis were measured before and after stimulation with LPS and incubation with selected antioxidants (vitamin C and NAC) in untreated HIV positive individuals. These levels were compared to a control group. We developed an assay that demonstrates the response of CD4+ T-cells to LPS-induced stimulation and, further, showed the inhibitory effect of antioxidants in this process.

## 2. Materials and Methods

### 2.1. Study Population and Design

In this cross-sectional study, twenty untreated, asymptomatic HIV positive individuals and 20 controls (32 females and 8 males) were sourced from a single HIV testing and prevention primary health clinic in Crossroads, Cape Town, South Africa. The median age of the participants was 32 years (range 22–42). There was no significant difference in age between the two groups. The HIV positive group had a significantly (*P* = 0.0003) lower CD4 count compared to the control group. The patients' demographics are summarised in Table 1. Informed consent was taken from all the participating subjects. Inclusion criteria for the study participants were 21 years or older, individuals with HIV infection and CD4 counts >200; not on antiretrovirals (ARVs) or any other chronic medication or antioxidant supplements. Exclusion criteria included patients with tuberculosis (TB) or other coinfections and those receiving antiretroviral therapy, anti-TB treatment or other antibiotic treatment, antioxidant supplementation, mineral and vitamins supplements, aspirin, or any other drug, for example, anti-inflammatory.

Ethics approval was obtained from both the clinical site, University of Cape Town: REC: REF: 417/2006 and laboratory site, University of Stellenbosch HREC N07/09/197. 

### 2.2. Reagents

Flow-check Fluorospheres, Flow-set Fluorospheres, CD4-PE, CD4-APC, CD25-PE, and annexin V-FITC/7AAD-PE kit were obtained from Beckman Coulter, Miami Florida Inc. (USA). FC 500 cytometer with two lasers, from Beckman Coulter, Miami, Florida, USA, FL, was used to acquire the data. L-ascorbic acid stock powder or vitamin C (C_6_H_8_O_6;_ molecular weight 176.12 g/mol; 25 g powder) and N-acetyl-L-cysteine stock powder (C_5_H_9_NO_3_S; 25 g powder) were purchased from Sigma-Aldrich (South Africa). 

### 2.3. Sample Preparation

Blood was drawn into two 10 mL tubes with Heparin, one 5 mL tube with EDTA (for viral load) and one 5 mL tube with citrate (for D-dimers). Samples were then couriered from the clinic to the laboratory within two hours of collection.

Heparinized whole blood samples were incubated with antioxidants for 20 min then stimulated with LPS, incubated overnight, and analysed on flow cytometer. Briefly, 100 *μ*L of blood was added into the labelled tubes and 30 *μ*L of vitamin C (10 mM) or 20 *μ*L of NAC (5 *μ*M) vortexed gently and incubated for 20 minutes. An additional tube was prepared with the “cocktail” of both antioxidants. After 20-minute incubation of the samples at 37°C with 5% carbon dioxide (CO_2_), 20 *μ*L of LPS (2 *μ*g/mL) was added. The dosages (2 *μ*g/mL LPS, 10 mM vitamin C, and 5 *μ*M NAC) used in this study were chosen for these experiments after a rigorous optimization study on the effects of temperature, time, and concentration on LPS-induced whole blood activation and antioxidant intervention in asymptomatic untreated HIV infection previously done in our lab (data not shown); these doses were chosen (data not shown). The samples were incubated overnight and analysed on flow cytometer.

For each sample, 100 *μ*L of blood was added to appropriately labelled Beckman's flow tubes and 10 *μ*L of monoclonal antibody mix was added. The sample was vortexed gently and incubated at room temperature for 15 minutes in the dark after which 500 *μ*L of fluorescein-activated cell sorting (FACS) lysing solution was added. The sample was vortexed gently and incubated for 15 minutes at room temperature. After incubation, 250 *μ*L of ice cold staining buffer and 250 *μ*L of binding buffer were added. The sample was spun at 300 g for 5 minutes after which 750 *μ*L of supernatant was removed. The pellets were resuspended, 200 *μ*L of staining buffer and and 200 *μ*L of binding buffers were added and analysed on flow cytometer. 

### 2.4. Flow Cytometry Analysis

An FC 500 flow cytometer (Beckman Coulter, Miami, FL, USA) with two lasers, five fluorescence channels, and CXP analysis software were used in this study. Alignment of the lasers was performed with a mixture of Flow-check and Flow-check beads. The appropriate voltages were determined and standardized with a mixture of Flow-set and Flow-set beads. Full matrix colour compensation was done using FITC, PE, APC, and PerCP/PC5 stained whole blood cells prepared using the lyse and wash method. A panel was created for test analysis using the cytometer settings established with flow-set and full matrix colour compensation. CXP and FCS Express V3 software programs were used to analyse the flow cytometry data.

### 2.5. Data Acquisition and Analysis for Apoptosis

**Figure 1:**
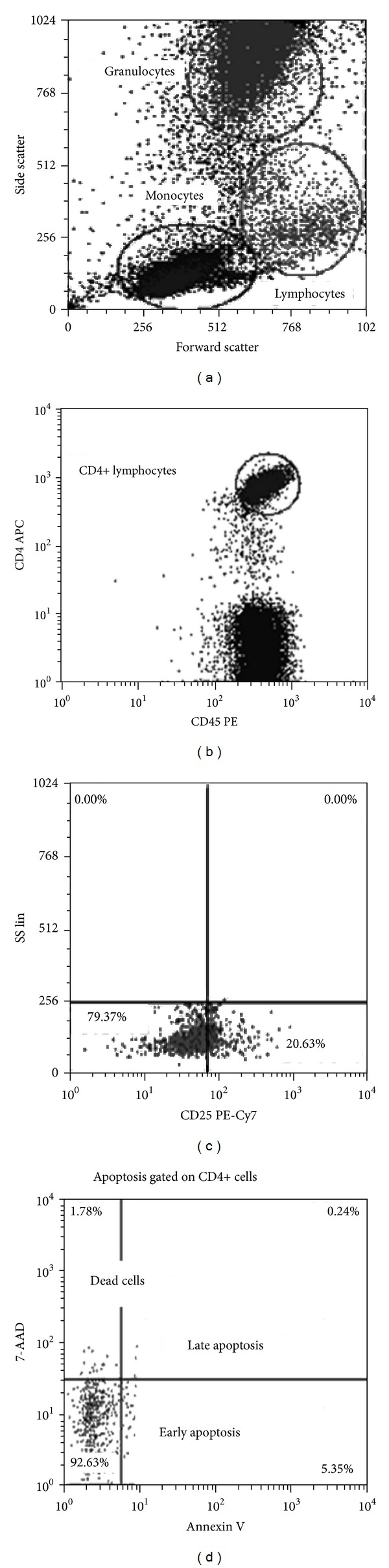
Gating strategy for activation (CD25) and apoptosis (annexin V/7-AAD). Plots (a) (side scatter versus forward scatter), (b) (bright CD4+ cells gated from the CD45+ leukocytes), (c) (CD25+ cells gated from the CD4 gate) and (d) (early, late apoptosis, and dead cells gated from CD4+ cells gate) show dot plots of whole blood used to set the quadrants.

For apoptosis, plot quadrants were set using unstained cells for every sample such that the negative annexin V cells and 7-AAD negative population lay in the first decade of the *Y* and *X* axis. A sequential gating strategy, by first gating on lymphocytes for CD4+ T-cells and then gating on CD4 T-cells for annexin V versus 7-AAD,was performed. This was in order to detect Annexin V + 7-AAD negative cells (apoptotic cells), Annexin V negative 7-AAD positive cells (dead cells), and Annexin V positive 7-AAD positive (secondary apoptotic or necrotic) CD4 T-cells as shown in Figure 1. A total of 300,000 events were acquired in order to analyse a minimum of 2000 CD4+ T-cells. CXP and FCS express V3 software programmes were used to analyse flow cytometry data.

### 2.6. Markers of Disease and Immune Activation

CD4 T-cell counts were determined by staining whole blood with Becton Dickinson (BD) MultiTest CD3-FITC/CD8-PE/CD45-PerCP/CD4-APC reagent in BD TruCount tubes according to the manufacturer's instructions and analysed on a BD FACSCalibur flow cytometer (BD Biosciences, San Jose, CA, USA). HIV-1 RNA quantifications were performed using 1.0 mL of plasma with the use of the Nuclisens Easy Q HIV-1 v.1.2 kit (BioMerieux Inc., Boxtel, the Netherlands). CD38 expression on CD8+ T-cells (CD38/8) was determined by flow cytometry. Whole blood samples were incubated with the monoclonal antibodies: CD8-Per-CP; and CD38-APC; CD3-FITC (BD Biosciences, San Jose, CA, USA) and analysed on a BD FACSCalibur instrument using BD Cell Quest Pro (Version 2) software. Lymphocytes were gated on forward versus side scatter, CD3, and CD8 expression.

### 2.7. D-Dimers

D-dimer, a marker of fibrinogen breakdown and clot formation, thus an indirect marker of inflammation, was determined by spectrophotometry using the IL-D-dimer method. This is an automated immunoassay for quantitative determination of D-dimers in plasma. Plasma from sodium citrate blood samples was mixed with latex reagent and buffer all supplied by Beckman Coulter (Miami, FL, USA) and agglutination, measured as decrease in absorbance, was read at 405 nm using ACL TOP from Beckman Coulter (Miami, FL, USA).

### 2.8. Statistical Analysis

The data was analysed using the Graphpad Prism version 5 statistical analysis software. Comparisons between the groups (HIV+ and HIV−) were done. Analysis of variance (ANOVA) was used to determine whether the means of the two groups (HIV+ and HIV−) differed significantly. Mann-Whitney nonparametric test and spearman's correlation were applied. Results were reported as medians with interquartile ranges. A 5% or lower significance level was used to determine significant findings (*P* ≤ 0.05). 

## 3. Results

### 3.1. Demographics of Study Population

The participants' demographics are summarized in Table 1. The group included 20 HIV positive and 20 controls most of whom were females (Fisher's test *P* = 0.36). There was no significant (*P* > 0.05) difference between the two groups in terms of age. Both groups had similar mean ages: 31 years for HIV positive group and 30 years for the control group. The HIV positive group had a significantly (*P* = 0.0012) lower CD4 count compared to the control group. The HIV positive group had a well-maintained CD4 count averaging 464 cells/mm^3^ (median-411 cell/mm^3^) and was clinically well. Median viral load was 45705 copies/mL. The control group had a high CD4 count with an average of 746 cells/mm^3^ and was also clinically well.

### 3.2. %CD25 Expression in the HIV Positive and Control Groups

**Figure 2:**
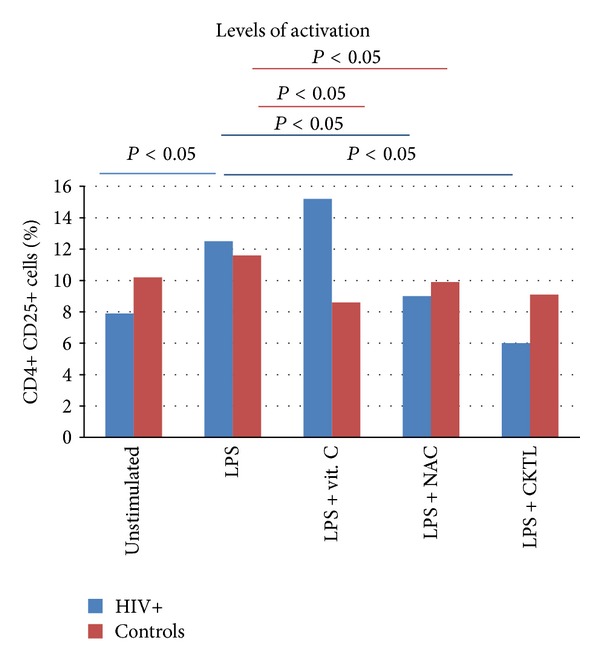
The figure shows the median %CD25 expression on CD4+ T cells under different experimental conditions for both HIV positive and control groups. LPS induced a significant increase in CD25 expression in HIV infection (*P* = 0.03) and this increase was similar to that of the controls (*P* = 0.68). Red and black lines at the top of the bars indicate significance.

The %CD25 expression before and after stimulation with LPS and incubation with vitamin C and NAC of the forty study participants is shown in Table 2 and illustrated in Figure 2. Baseline (unstimulated) levels of activation were not significantly different between the two groups (*P* = 0.40), however, after stimulation, the HIV positive group showed statistically significant increase in activation (*P* = 0.03) when compared to the controls, which was not significant (*P* = 0.16). A significant difference was noted with the incubation of LPS and vitamin C alone and NAC alone: in the control group. Optimal levels of inhibition of activation in the HIV group were achieved with the combination of NAC + vitamin C (*P* = 0.0018).

### 3.3. The % Annexin V/7-AAD Staining for Early and Late Apoptosis between the HIV Positive and Control Groups

**Figure 3:**
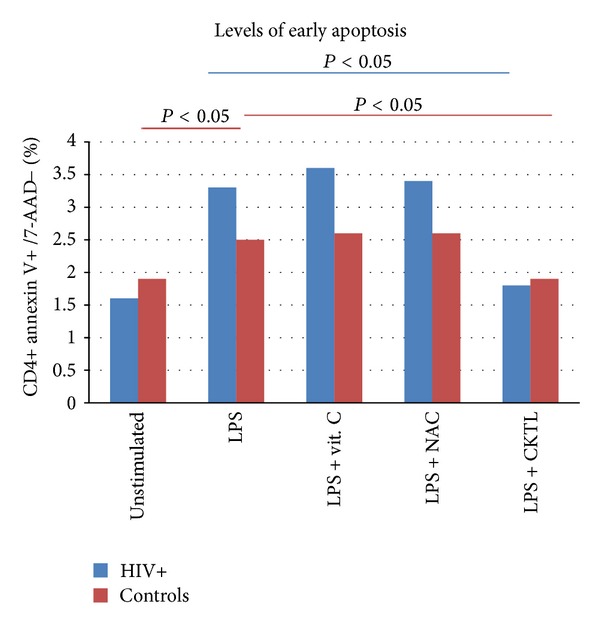
The figure shows the median % annexin V staining on CD4+ T-cells under different experimental conditions for both HIV positive and control groups. LPS induced a significant increase in annexin V staining in the controls (*P* = 0.007) and this increase was significantly reduced in both HIV positive and controls (*P* = 0.02; *P* = 0.008, resp.) by a combination of vitamin C and NAC. Red and blue lines at the top of the bars indicate significance.

**Figure 4:**
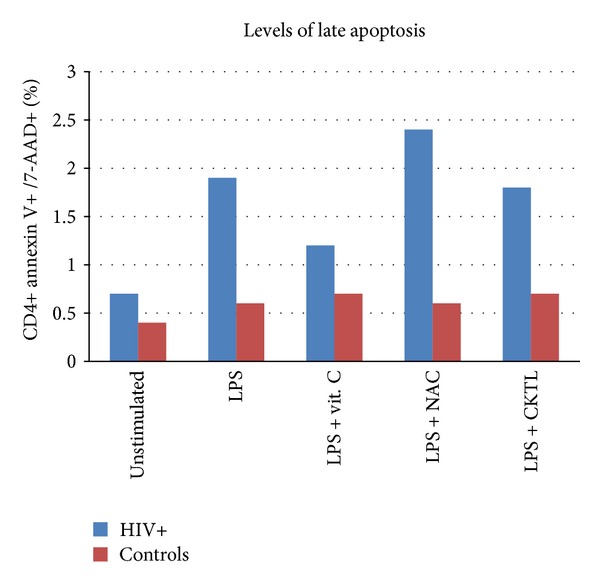
The figure shows the median % annexin V+/7-AAD+ staining on CD4+ T-cells under different experimental conditions for both HIV positive and control groups. LPS did not induce a significant increase in annexin V+/7-AAD+ staining in both groups (*P* > 0.05).

The % Annexin V/7-AAD staining before and after stimulation with LPS and overnight incubation with vitamin C and NAC is summarised in Tables 3 and 4, and Figures 3 and 4. For early apoptosis, at baseline, the levels of annexin V+/7AAD-staining were not significantly (*P* > 0.05) different between the two groups; however, a significant difference was noted after stimulation with LPS (*P* = 0.007) in the control group. There was a significant difference after incubation with LPS and a combination of NAC and vitamin C in both groups. Importantly, the combination of vitamin C and NAC significantly (*P* < 0.0001) reduced the annexin V+/7-AAD- staining cells back to its unstimulated levels. NAC and vitamin C in combination significantly (*P* = 0.007, *P* = 0.002) decreased the staining of annexin V+/7-AAD-cells. For late apoptosis, there was no significant difference (*P* > 0.05) before stimulation with LPS and after stimulation with LPS and incubation with the antioxidants individually or in combination, meaning that these antioxidants are effective in limiting early apoptosis. This could help in retaining their functionality and protecting them from early death.

### 3.4. Other Markers of Disease in HIV Infection

Table 5 shows the values of other markers of disease and immune activation in the cohort. When compared with the controls as expected, the HIV positive individuals had significantly (*P* < 0.05) lower CD4 counts. D-dimers, an indirect (marker of fibrinogen breakdown and clot formation) marker of inflammation, was significantly (*P* < 0.05) higher in HIV positive patients than in controls. 

There was a strong inverse correlation between CD4 count and viral load (*r* = −0.62; *P* = 0.03) and negative correlation between CD4 count and %CD38/CD8+ (*r* = −0.48; *P* = 0.05). However, there was no correlation between CD4 counts and D-dimers.

## 4. Discussion

Persistent immune activation characterises HIV infection and is associated with the depletion of CD4+ T-cells, increased risk of disease progression, and higher mortality. The breakdown of gut mucosal integrity in early HIV infection results in translocation of microbial products such as lipopolysaccharide (LPS) into the systemic circulation. This is an important source of on-going immune stimulation. In this study, therefore, we developed an assay to determine the ability of CD4+ T-cells in HIV to be activated by LPS *in vitro* and further to be inhibited by selected antioxidants. 

LPS induced a significant increase in CD25 (activation marker) expression in HIV infection when compared to that of the controls (*P* = 0.68). Thus, at this stage of the infection, with relatively well-maintained CD4 counts and no clinical symptoms, CD4 T-cells in HIV infection retain the ability to be activated, which was significantly reduced by NAC and a combination of NAC and vitamin C. After incubation with antioxidants and stimulation with LPS, interestingly, the HIV positive group showed good responses to NAC alone and a “cocktail” of NAC and vitamin C, with CD25 levels returning to below baseline values, however, a similar effect could only be demonstrated in the control group with vitamin C alone. The combination of vitamin C and NAC was required to achieve optimal inhibition of the LPS-induced-activation. 

LPS induces activation of innate immune cells such as monocytes and dendritic cells resulting in increased production of proinflammatory cytokines consequently inducing oxidative stress. This leads to depletion of antioxidant defence mechanisms and an increased susceptibility to apoptosis. CD25 is a relatively late marker of activation [16]. In the study by Tincati et al. on the effects of *in vitro *LPS stimulation on T-cells in patients on HAART, significantly higher CD4+ and CD8+ expressing HLA-DR and CD38+ expressing cells were detected in low and intermediate responders compared to the HIV negative group confirming a sustained immune activation in HIV infection [15]. The current study confirms Tincati et al. findings of increased LPS-induced immune activation as measured by CD25 expression in HIV infection when compared to controls. Furthermore, there was a significantly (*P* < 0.05) increased immune activation expressed as CD38 in the HIV positive group when compared to the controls.

Annexin V staining was also significantly increased after stimulation with LPS in HIV positive and control groups but was more pronounced in the HIV positive group. NAC alone and in combination with vitamin C significantly reduced early apoptosis of CD4+ T-cells to a greater degree than vitamin C alone. Moreover, LPS induced a significant increase in 7-AAD staining in HIV infection, which was significantly reduced by the antioxidants either alone or in combination. 

This study demonstrates that LPS was capable of inducing CD4 T-cell activation and apoptosis *in vitro *as indicated by increased CD25, annexin V, and 7-AAD, which was ameliorated by the combination of antioxidants. In addition, NAC alone significantly reduced LPS-induced activation and apoptosis of CD4+ T-cells in HIV infection. Early work demonstrated that NAC administration to HIV positive individuals was able to slow down CD4 decline in HIV infection [17]. NAC and glutathione have been shown to completely block activation-induced death and associated DNA fragmentation in T-cell hybridomas, therefore implicating redox regulation in the processes [18]. In addition, NAC has been shown to directly scavenge free radicals by decreasing hypochlorous acid produced by neutrophils [19, 20]. Cell studies have indicated that NAC enhances intracellular killing of bacteria by protecting the neutrophils and macrophages from free radicals generated during phagocytosis [21]. In support of the current study findings, NAC has been shown previously to inhibit LPS-mediated activation; however, this work was performed in rats kupffer cells [14]. The effect of NAC on T-cell activation and apoptosis particularly in the context of HIV infection is not well documented and therefore the findings of this study may be of value in the future management of persons living with HIV.

An important finding of this study was that vitamin C had no effect on LPS-induced activation when used alone in HIV, but when used in combination with NAC (cocktail), it showed a significant reduction in CD4+ T-cell activation levels. Previous studies have reported clinical improvement in AIDS patients who willingly consumed high doses (500 mg, 800 mg and 1 800 mg) of ascorbic acid [22–25]. In addition vitamin C has been shown to inhibit NF-*κ*B activation via multiple stimuli including IL-1 and TNF in endothelial cell lines ECV30VS and in primary HUVECS [26]. Several studies have shown that vitamin C inhibited T-cell pathways of apoptosis which includes the upregulation of the antiapoptotic B-cell lymphoma-2 (Bcl-2) protein expression levels [27–29]. Although vitamin C exhibits strong antioxidant properties, it has been demonstrated *in vitro* that it can also act as a prooxidant in the presence of free transition metals [30, 31]. In this setting, it generates hydroxyl radicals in a fenton-like reaction. This could explain the cause of activation of CD4+ T-cells when vitamin C was used alone in the current study. In support of this, Bergman et al. demonstrated a 39% increase in apoptotic cells when cells were incubated with 0.2 mg/mL vitamin C for 24 hrs [32]. Previous data on clinical trials using vitamin C have been conflicting. Some authors have suggested that supplementation with vitamin C is toxic [33, 34]. In this study, only low concentrations (10 mM) of vitamin C were utilized. Previous work in our laboratory (results not shown) had demonstrated that higher concentrations were toxic and able to cause activation and even death of the cells. Thus, at higher doses, vitamin C is likely to have a prooxidant effect which causes activation and even death of cells.

Therefore, the current study has developed an important assay that demonstrates the response of CD4+ T-cells to LPS-induced stimulation and further showed the effects of antioxidants in this process. The study was able to demonstrate that at this stage of HIV infection, CD4+ T-cells were able to respond to LPS-induced stimulation and antioxidants; therefore, they do not appear “exhausted” at this stage of the disease. However, it should be noted that LPS induced more death in the form of annexin V+/7-AAD+ staining in the HIV group than the control, suggesting that the cells may have been “primed” for death previously *in vivo. *Vitamin C alone did not inhibit LPS-induced activation in the HIV group as it did in the controls; suggesting that the use of vitamin C alone in HIV infection would not be of value. The combination of NAC with vitamin C produced the greatest level of inhibition of early apoptosis, suggesting a potential beneficial effect of this cocktail in the management of this stage of the infection. It is at this early stage of HIV infection that the “cocktail” is being most effective and this study has demonstrated beneficial effects of the cocktail in limiting immune activation and early apoptosis. This way immune cells can be rescued before irreversible damage to the cells occurs.

Limitations of the study were that specific tests for diagnosing underlying subclinical infections could not be performed and smoking and alcohol habits were not documented. Longitudinal cohort studies will be important to determine the value of intervention with the combination of anti-oxidants as described in this study, in HIV positive persons with CD4 counts >350 cells/mm^3^.

## 5. Conclusion

This is an important assay that demonstrated the response of CD4+ T-cells to LPS-induced stimulation and showed the inhibitory effects of antioxidants. Certain combinations of antioxidants could be important in reducing the harmful effects of chronic immune activation and thereby limit CD4+ T-cell depletion. Importantly, the study showed that CD4+ T-cells of the HIV positive group responded better to a combination of vitamin C and NAC. Therefore, appropriate intervention at this asymptomatic stage could rescue the cells before exhaustion and senescence set in.

## Figures and Tables

**Table 1:** Demographics characteristics of both the HIV positive and control groups.

Parameter	HIV positive group (*n* = 20)	Controls (*n* = 20)	*P* values
Male : female	5 : 15	3 : 17	
Median age (yrs)	31.8 (27–35)	30.3 (22–35)	0.43
Range	21–51	21–48	
Median CD4 cells/mm^3^	411 (265–634)	753 (564–870)	0.0012**
Median viral load (copies/mL)	45705 (2174–157294)	ND	
Log viral load	4.0 (3.1–5.4)		

All the values in columns are median (interquartile range) of HIV positive (*n* = 20) individuals and controls (*n* = 20). **Significant at *P* = 0.05.

**Table 2:** The %CD25 expression before and after overnight stimulation with LPS and incubation with vitamin C and/or NAC.

% CD25	HIV+ group	Controls	*P* value between HIV and control	*P* value for LPS activation and inhibition by antioxidants in HIV group	*P* value for control
Unstimulated	7.9 (7.1–14.4)	10.2 (7.8–14.4)	0.4047		
LPS stimulated	12.5 (10.3–17.6)	11.6 (9.6–15.2)	0.6823	0.0337**	0.16
LPS + Vit C	15.2 (11.7–17.6)	8.6 (7.2–11.9)	0.0003**	0.4	0.01**
LPS + NAC	9.0 (7.1–14.2)	9.9 (6.9–13.1)	0.9033	0.0416**	0.07
LPS + cocktail	6.0 (4.2–13.1)	9.1 (7.8–14.6)	0.0501	0.0018**	0.22

All the values are median (interquartile range) percentages of cells expressing CD25 of the HIV positive and control groups, unstimulated and stimulated with LPS and incubated overnight. **Medians significant at *P* < 0.05.

**Table 3:** The % annexin V+/7-AAD− staining of the HIV positive and the control groups.

% Annexin V	HIV positive group	Control	*P* value (HIV and controls)	*P* value for LPS activation and inhibition by antioxidants in HIV+ group	*P*-value for controls
Unstimulated	1.6 (0.9–4)	1.9 (1.2–2.3)	0.49		
LPS-activated cells	3.3 (1.6–5.6)	2.5 (2.0–3.2)	0.17	0.11	0.007**
LPS + VIT C	3.6 (1.9–4.2)	2.6 (2.1–3.4)	0.26	0.84	0.44
LPS + NAC	3.4 (1.2–4.3)	2.6 (2.2–3.4)	0.55	0.54	0.66
LPS + CKTL	1.8 (1.0–3.7)	1.9 (1.5–2.4)	0.96	0.02**	0.008**

All the values are median (interquartile range) percentages of cells staining with annexin V/7-AAD− of the HIV positive and control groups, unstimulated and stimulated with LPS and incubated overnight. *Medians are marginally significant at *P* = 0.05. **Medians significant at *P* < 0.05.

**Table 4:** The % annexin V+/7-AAD+ staining of the HIV positive and HIV negative groups.

%7-AAD	HIV positive group	Control	*P* value between HIV and control	*P* value for LPS activation and inhibition by antioxidants in HIV group	*P* values for the controls
Baseline	0.7 (0.4–2.2)	0.4 (0.3–0.5)	0.02**		
LPS-activated cells	1.9 (0.7–3.2)	0.6 (0.4–0.7)	0.0029**	0.10	0.14
LPS + VIT C	1.2 (0.7–2.7)	0.7 (0.4–1.0)	0.0048**	0.89	0.27
LPS + NAC	2.4 (1.0–3.4)	0.6 (0.4–0.8)	0.0003**	0.64	0.79
LPS + CKTL	1.8 (1.3–2.1)	0.7 (0.6–1.2)	0.0001***	0.76	0.56

The values are median (interquartile range) percentages of cells staining for both annexin V and 7-AAD of the HIV positive and control groups, unstimulated and stimulated with LPS and incubated overnight. **Medians significant at *P* < 0.05. ***Medians significant at *P* ≤ 0.0001.

**Table 5:** Other markers of disease in HIV infection in both the HIV positive and control groups.

	HIV positive group	Control group	*P* value
D-dimers (mg/L)	0.23 (0.2-0.3)	0.21 (0.20–0.26)	<0.0001**
CD38/8 (%)	27.6 (17.5–44.0)	11.6 (7.3–15.8)	<0.0001**

The table shows the D-dimers and %CD38/8 in these groups expressed as median (interquartile range). **indicates that the medians were significantly different at *P* = 0.05. CD38/8 was significantly different (*P* < 0.0001).
